# Effect of low thermal pasteurization in combination with carvacrol on color, antioxidant capacity, phenolic and vitamin C contents of fruit juices

**DOI:** 10.1002/fsn3.611

**Published:** 2018-03-06

**Authors:** Alex Tchuenchieu, Jean‐Justin Essia Ngang, Marjorie Servais, Michael Dermience, Sylvain Sado Kamdem, François‐Xavier Etoa, Marianne Sindic

**Affiliations:** ^1^ Department of Microbiology University of Yaoundé I Yaoundé Cameroon; ^2^ Analysis, Quality and Risk Unit Gembloux Agro‐Bio Tech‐University of Liège Gembloux Belgium; ^3^ Centre for Food and Nutrition Research IMPM Yaoundé Cameroon

**Keywords:** carvacrol, color, fruit juices, mild heat, nutritional value

## Abstract

Mild thermal treatment in combination with natural antimicrobials has been described as an alternative to conventional pasteurization to ensure fruit juices safety. However, to the best of our knowledge, no study has been undertaken to evaluate what could be its effect on their color and nutritional value. This study therefore aimed at assessing how a low thermal pasteurization in combination with carvacrol could affect these parameters, with orange, pineapple, and watermelon juices as selected fruit juices. The experimental design used had levels ranging from 50 to 90°C, 0 to 60 μl/L, and 0 to 40 min for temperature, concentration of carvacrol supplemented, and treatment length, respectively. The only supplementation of fruit juices with carvacrol did not affect their color. In comparison with high thermal pasteurization (>70°C), a combined treatment at mild temperatures (50–70°C) better preserved their color, antioxidant capacity (AOC), and vitamin C content, and increased their total phenolic content (TPC). Globally, carvacrol supplementation had a positive impact on the TPC of thermally treated juices and increased the AOC of treated watermelon juice, which was the lowest of the three fruit juices. Mild heat treatment in combination with natural antimicrobials like carvacrol is therefore an alternative to limit the negative effects of conventional pasteurization on fruit juices quality.

## 1 INTRODUCTION

Fruit juices are considered as important components of a healthy diet, and their regular intake is highly encouraged and recommended. They contain low levels of fat and high levels of vitamins, minerals, and dietary fiber. They are major sources of vitamin C and have a high antioxidant capacity (AOC) which helps fight against stress and prevents cardiovascular diseases, hypertension, atherosclerosis, diabetes, and cancers (Aneja, Dhiman, Aggarwal, & Aneja, 2014; Gardner, White, McPhail, & Duthie, 2000; Paul & Ghosh, 2012; Vikram, Ramesh, & Prapulla, 2005). Their consumption has increased in recent years due to promotion of their natural flavor, nutritional quality, and various health benefits (Aneja, Dhiman, Aggarwal, Kumar, & Kaur, 2014).

A number of foodborne outbreaks have been associated with the consumption of nontreated fruit juices (Aneja, Dhiman, Aggarwal, & Aneja, 2014; Aneja, Dhiman, Aggarwal, Kumar, et al., 2014; Vojdani, Beuchat, & Tauxe, 2008), that is why fruit juices production process usually integrates a pasteurization step in order to ensure their microbial safety and stability. However, the intensity of the heat treatment generally applied deteriorates the nutritional quality of fruit juices and modify their physicochemical and sensorial properties (Aneja, Dhiman, Aggarwal, Kumar, et al., 2014; Chen, Yu, & Rupasinghe, 2013; Jiménez‐Sánchez, Lozano‐Sánchez, Segura‐Carretero, & Fernández‐Gutiérrez, 2015a,2015b). These limitations have stimulated the research and the development of treatments with a minimal effect on the above properties of fruit juices (Cortés, Esteve, & Frígola, 2008). Moreover, consumer demand for high‐quality, minimally processed, and microbiologically safe foods is increasing. Several low‐temperature technologies have been proposed, including irradiation, high hydrostatic pressure, pulsed electric fields, and ultraviolet (Chen et al., 2013; Jiménez‐Sánchez et al., 2015a,2015b; Rupasinghe & Yu, 2012). Unfortunately, the high initial investment required to acquire the technical equipment has limited their widespread use (Espina, Somolinos, Pagán, & García‐Gonzalo, 2010).

In a hurdle strategy approach, many studies have shown that mild heat treatment in combination with natural antimicrobials, especially plant‐derived ones (essential oils or their pure aroma compounds), is a potential alternative to ensure the safety of fruit juices (Ait‐Ouazzou, Espina, García‐Gonzalo, & Pagán, 2013; Aneja, Dhiman, Aggarwal, Kumar, et al., 2014; Belletti, Kamdem, Tabanelli, Lanciotti, & Gardini, 2010; Belletti et al., 2007; Char, Guerrero, & Alzamora, 2009; Espina, Condón, Pagán, & García‐Gonzalo, 2014; Espina et al., 2010, 2012; Kamdem, Belletti, Magnani, Lanciotti, & Gardini, 2011; Ngang et al., 2014). In fact, these specific natural antimicrobials are generally recognized as safe (GRAS) (Burt, 2004; Calo, Crandall, O'Bryan, & Ricke, 2015; Huertas, Esteban, Antolinos, & Palop, 2014; Lucera, Costa, Conte, & Del Nobile, 2012). However, the positive impact of this hurdle process on the nutritional value of fruit juices has always been hypothesized, but not studied. Furthermore, no study has been carried out on the final appearance of the treated juices, the latter being an important quality indicator for consumers.

Carvacrol (5‐isopropyl‐2‐methylphenol) is the major component of *Origanum* spp essential oil. It is a GRAS compound known for its strong and wide antimicrobial spectrum and classified as a natural economical food preservative (Nostro & Papalia, 2012; Ramos et al., 2013; Sánchez, Aznar, & Sánchez, 2015). Kamdem et al. (2011) and Ait‐Ouazzou et al. (2013) have observed that mild thermal treatment of fruit juices in combination with this compound could be a method to ensure safety against *Listeria monocytogenes* 56 LY and *Escherichia coli* O157:H7. This study aimed at assessing how such combined treatment could affect visual color and nutritional value of some fruit juices.

## 2 MATERIALS AND METHODS

### 2.1 Fruit juices production

Oranges (*Citrus sinensis*), pineapples (*Ananas comosus*), and watermelons (*Citrullus vulgaris*) were purchased from a local supermarket of Gembloux (Belgium). The juices were extracted using a fruit juice extractor (Kenwood A900 Centrifuge, Tokyo, Japan) and passed through a filter with a pore diameter of 280 μm. The pH and Brix values of the produced juices were measured using a portable pH meter ProfiLine pH 3210 Set 4 (WTW GmbH, Weilheim, Germany) and an Atago DBX‐55 refractometer (Atago Co. Ltd., Tokyo, Japan), respectively.

### 2.2 Experimental design

A central composite design (CCD), reinforced at its center and edges with three variables at five equidistant levels, was implemented. Hue angle value, AOC, total phenolic content (TPC), and vitamin C or ascorbic acid content (AAC) of the juices after treatment were measured to assess the effects of treatment temperature, supplemented concentration of carvacrol (natural, 99%, Food Grade, Sigma‐Aldrich, St. Louis, MO, USA), and duration of the thermal treatment. The tested levels of temperature (50–90°C) and length of treatment (0–40 min) were chosen to cover those generally tested in combination with natural antimicrobials and those conventionally practiced in fruit juice industries. The concentrations of carvacrol to be tested (0–60 μl/L) were chosen based on a preliminary sensorial analysis (Tchuenchieu et al., 2018). Each of the 27 runs of the CCD was performed in triplicate. The effect of the treatment on the AAC was assessed only with orange juice as this juice is known to have a higher content compared to the two other fruit juices.

Besides, the change in color and the variation of AOC and TPC of juices induced by the only carvacrol supplementation at the tested concentrations were also assessed.

### 2.3 Thermal treatment

**Figure 1:**
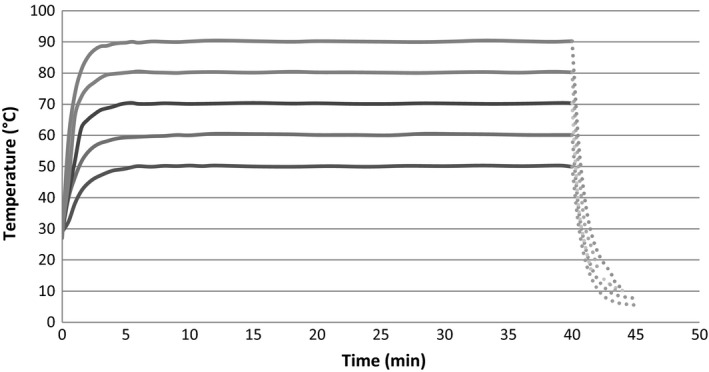
Overview of the temperature profiles during the treatments of juice samples at the different tested temperatures in water bath (continuous lines) and once in the cryogenic solution (discontinuous lines)

Thermal treatment was performed as described in a previous study (Ngang et al., 2014). It consisted of vials introduced in a thermostatically controlled water bath with fixed temperature to attain the desired temperature in the juice samples. For each tested condition, vials containing 9.9 ml of juice were supplemented with 0.1 ml of an ethanolic solution of carvacrol. The samples were then heated, and length of treatment measured from the moment the juice reached the desired temperature. (Generally, it took about 5 min to reach this temperature.) The samples were immediately cooled in a cryogenic solution (−10°C) once the treatment length was reached. Temperature profiles during the thermal treatments (Figure 1) were followed using an electronic thermometer (TESTO 905‐T1, Kanagawa, Japan).

### 2.4 Color measurement

The hue angle values of the juice samples, which characterize their visual color, were measured using a ColorFlex EZ colorimeter (Hunter Associates Laboratory Inc., VA, USA). The instrument was calibrated and used according to the manufacturer's recommendations.

### 2.5 Measurement of antioxidant capacity

Antioxidant capacity (AOC) was measured using the DPPH (1,1‐diphenyl‐2‐picrylhydrazyl) method (Plaza et al., 2006). Samples were diluted fourfold with methanol. Then, 0.2 ml of the diluted sample was added to 7.8 ml of a 0.03 g/L methanolic solution of DPPH (Sigma‐Aldrich). The obtained mixture was then shaken on a vortexer and incubated in the dark for 30 min before reading the absorbance at 515 nm with a UV‐2401 spectrophotometer (Shimadzu, Kyoto, Japan). Trolox (6‐Hydroxy‐2,5,7,8‐tetramethylchromane‐2‐carboxylic acid) from Sigma‐Aldrich was used as standard. The calibration curve was plotted using concentrations from 0 to 2 mmol/L. The AOC of samples was expressed as mmol/L TEAC (Trolox Equivalent Antioxidant Capacity).

### 2.6 Measurement of total phenolic content

Total phenolic content (TPC) of the samples was determined by the Folin–Ciocalteu method (Medina, 2011). Samples were first diluted fourfold with distilled water, and a volume of 0.5 ml of the diluted juice was then added to 4.3 ml of distilled water and 0.2 ml of Folin–Ciocalteu 2 N (Sigma‐Aldrich). After vortexing, 0.5 ml of a 20% Na_2_CO_3_ solution and 4.5 ml of distilled water were added. The mixture was then vortexed again and incubated in the dark for 1 hr at room temperature before reading the absorbance at 725 nm. Gallic acid (Sigma‐Aldrich) was used as standard. The calibration curve was plotted using concentrations from 0 to 500 mg/L. The TPC of samples was expressed as mg/L gallic acid equivalent (mg/L GAE).

### 2.7 Measurement of ascorbic acid content

Ascorbic acid content (AAC) was measured by polarography using a 626 Polarecord equipped with a 663 VA stand from Metrohm (Herisau, Switzerland). The electrode system consisted of a glass carbon auxiliary electrode, a dropping mercury electrode, and a silver chloride reference electrode (Ag/AgCl/KCl 3 mol/L). We added 10 ml of an acetate buffer solution (acetic acid–sodium acetate 0.5 N) to 10 ml of the juice sample. The mixture was poured into the polarographic vessel and deaerated with nitrogen gas for 5 min before analysis. The potential was ramped from +0.31 V to −0.15 V with a natural mercury drop. The calibration curve was plotted with L(+)‐ascorbic acid (VWR, Fontenay‐sous‐Bois, France) concentrations from 0 to 200 mg/L.

### 2.8 Statistical analysis

Statistical analyses were performed using Statistica.10 software of Statsoft (Dell, TX, USA). A one‐way ANOVA–Bonferroni post hoc test (homogeneous group) was performed to evaluate significant differences between the mean values obtained. Besides, an ANOVA–effect estimates analysis was also performed on the CCD results to assess the effect of the tested variables on each response studied.

## 3 RESULTS

### 3.1 Physicochemical characteristics of fruit juices and effect of carvacrol supplementation

The freshly squeezed orange, pineapple, and watermelon juices had a pH and Brix values of 3.7 ± 0.2 and 11.9 ± 0.3; 3.5 ± 0.1 and 14.7 ± 0.2; 5.5 ± 0.1 and 9.2 ± 0.2, respectively (*n* = 5).

**Figure 2:**
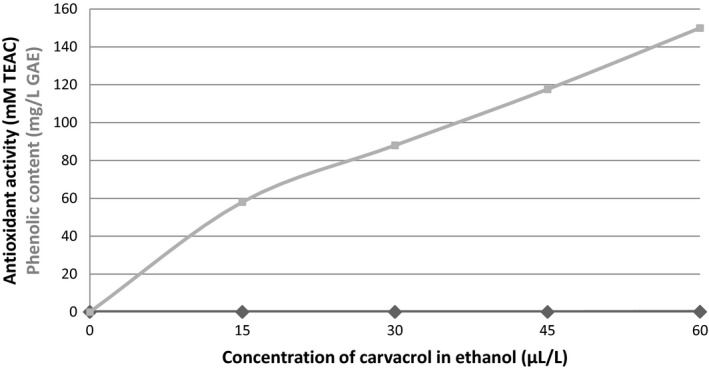
Antioxidant capacity (black lozenges) and phenolic content (gray squares) of carvacrol at the tested concentrations

**Figure 3:**
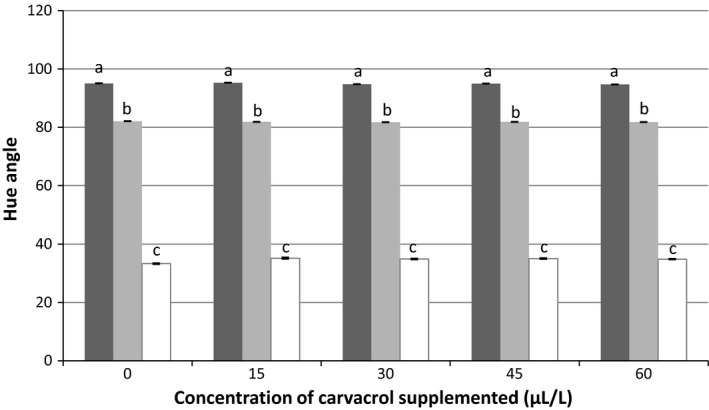
Hue angle values of pineapple (in black), orange (in gray), and watermelon (in white) juices when supplemented with carvacrol at the tested concentrations

As noticed with ethanolic solutions (Figure 2), the AOC of the tested concentrations of carvacrol (0–60 μl/L) was almost null, while their TPC was rising with the concentration supplemented. Supplementation of this compound to fruit juices up to 60 μl/L did not significantly affect their color (Figure 3). In fact, whatever the fruit juice, the hue angle values observed in the presence of carvacrol were similar to those of nonsupplemented samples.

### 3.2 Effect of combined treatment on the color and bioactive compounds of fruit juices

**Figure 4:**
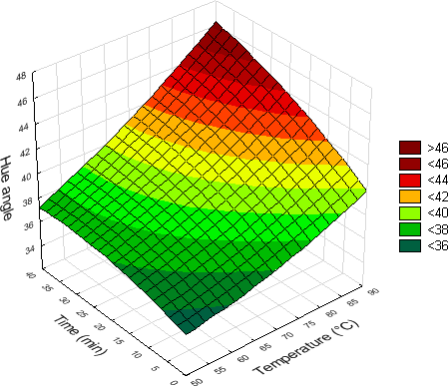
Effect of temperature and length of treatment on the visual color of watermelon juice (carvacrol = 30 μl/L)

Table 1 shows the hue angle values and change in the AOC of fruit juices after the different treatments. As revealed by the statistical analysis of these data (Table 2), temperature, length of treatment, and carvacrol supplementation had a significant effect on the hue angle values of the different juices. However, considering the coefficients characterizing their effects, their impact was almost null on the color of orange and pineapple juices. With watermelon juice, it is a significant high linear positive effect of temperature and length of treatment that was noticed. In fact, as illustrated by Figure 4, an important increase in the hue angle value of this juice was observed with the rise of temperature and length of treatment.

**Table 1:** Hue angle values and change in the antioxidant capacity of fruit juices after the different treatments

Treatment conditions			Hue angle value			Variation of the AOC (%)		
Temperature (°C)	Carvacrol (μl/L)	Time (min)	Pineapple juice	Orange juice	Watermelon juice	Pineapple juice	Orange juice	Watermelon juice
90	0	0^a^	97.33 ± 0.04	81.16 ± 0.02	38.15 ± 0.06	−13.95 ± 4.50	−7.11 ± 5.56	−31.04 ± 1.17
90	0	20	96.16 ± 0.03	81.30 ± 0.03	43.52 ± 0.14	−13.75 ± 3.03	−3.91 ± 3.66	−41.18 ± 7.26
90	30	20	95.65 ± 0.04	81.46 ± 0.03	43.73 ± 0.08	−13.75 ± 3.67	−10.01 ± 7.27	−34.86 ± 4.85
90	0	40	95.51 ± 0.06	80.50 ± 0.05	44.99 ± 0.01	−14.40 ± 3.10	−13.97 ± 7.03	−39.95 ± 6.17
90	60	40	95.54 ± 0.03	80.11 ± 0.03	46.45 ± 0.06	−12.22 ± 5.49	−7.16 ± 3.74	−30.03 ± 7.68
80	15	10	96.16 ± 0.04	81.86 ± 0.04	41.08 ± 0.02	−8.37 ± 3.05	−3.25 ± 5.46	−33.88 ± 7.97
80	45	10	96.20 ± 0.01	81.53 ± 0.04	40.99 ± 0.03	−12.75 ± 3.55	−2.59 ± 5.57	−30.58 ± 6.56
80	15	30	95.51 ± 0.03	82.11 ± 0.02	42.46 ± 0.06	−11.16 ± 3.10	−1.42 ± 7.87	−27.69 ± 10.32
80	45	30	95.80 ± 0.03	81.66 ± 0.03	42.65 ± 0.03	−8.80 ± 3.85	−5.49 ± 1.90	−21.90 ± 7.54
70	0	0^a^	97.01 ± 0.04	82.10 ± 0.02	36.32 ± 0.10	−4.30 ± 4.35	−7.20 ± 4.98	+3.31 ± 11.59
70	30	0^a^	97.21 ± 0.01	82.11 ± 0.02	36.74 ± 0.09	−1.94 ± 3.70	+1.78 ± 7.12	+18.07 ± 9.23
70	0	20	96.43 ± 0.02	82.39 ± 0.02	38.59 ± 0.04	−7.50 ± 2.27	−3.64 ± 6.66	+14.25 ± 6.49
70	30	20	96.35 ± 0.03	82.18 ± 0.04	39.56 ± 0.13	−6.42 ± 3.03	+5.60 ± 5.79	+23.16 ± 15
70	30	20	96.12 ± 0.04	82.35 ± 0.04	38.62 ± 0.12	−11.28 ± 4.61	+4.98 ± 15.88	+21.37 ± 9.00
70	30	20	96.06 ± 0.02	82.11 ± 0.01	39.35 ± 0.03	−3.99 ± 2.73	−0.36 ± 6.16	+28.75 ± 8.44
70	60	20	96.55 ± 0.03	82.25 ± 0.01	40.05 ± 0.03	−7.39 ± 2.51	+9.87 ± 6.13	+30.53 ± 5.96
70	0	40	95.52 ± 0.01	82.51 ± 0.06	40.54 ± 0.06	−4.23 ± 3.64	0.00 ± 4.36	+26.72 ± 8.80
70	30	40	95.55 ± 0.01	82.12 ± 0.04	42.17 ± 0.04	−4.17 ± 3.85	+6.67 ± 5.47	+22.90 ± 7.00
60	15	10	96.57 ± 0.01	82.08 ± 0.04	37.13 ± 0.34	−5.66 ± 4.36	−9.60 ± 3.66	+25.21 ± 10.15
60	45	10	96.54 ± 0.03	81.85 ± 0.01	37.57 ± 0.23	−8.67 ± 4.98	−0.83 ± 3.76	+34.71 ± 9.39
60	15	30	96.50 ± 0.05	82.73 ± 0.03	38.62 ± 0.04	−6.50 ± 3.55	−4.58 ± 5.34	+29.75 ± 11.52
60	45	30	96.27 ± 0.03	82.14 ± 0.02	38.45 ± 0.19	−5.47 ± 3.21	−4.04 ± 4.28	+39.67 ± 12.04
50	0	0^a^	95.89 ± 0.02	81.87 ± 0.05	34.51 ± 0.22	−5.55 ± 4.54	−1.17 ± 4.18	+19.01 ± 10.59
50	0	20	96.88 ± 0.02	81.95 ± 0.03	36.47 ± 0.31	−4.68 ± 6.07	−4.29 ± 3.93	+49.17 ± 13.02
50	30	20	96.77 ± 0.01	81.92 ± 0.02	37.29 ± 0.21	−4.74 ± 6.34	+2.14 ± 7.76	+68.60 ± 8.68
50	0	40	96.71 ± 0.04	82.05 ± 0.01	36.18 ± 0.17	−2.92 ± 2.44	−0.49 ± 5.07	+66.94 ± 17.72
50	60	40	97.07 ± 0.02	81.99 ± 0.02	37.29 ± 0.29	+1.46 ± 0.82	+5.95 ± 7.56	+81.40 ± 7.05
**Nontreated juice**			**Hue angle value**			**AOC (mmol/L TEAC)**		
			95.33 ± 0.02	81.97 ± 0.04	35.21 ± 0.12	4.38 ± 0.77	2.29 ± 0.68	0.42 ± 0.14

TEAC, Trolox Equivalent Antioxidant Capacity; AOC, antioxidant capacity.

a Samples were removed from the water bath as soon as they reached the desired temperature.

**Table 2:** Effect estimates of process variables on the change in hue angle values and antioxidant capacity of fruit juices observed

Factor	Effect on hue angle			Effect on antioxidant capacity		
	Pineapple juice	Orange juice	Watermelon juice	Pineapple juice	Orange juice	Watermelon juice
(1) Temperature (L)	−0.31^a^	−0.47^a^	3.41^a^	−4.81^a^	−3.35^a^	−48.89^a^
Temperature (Q)	0.04	−0.4^a^	0.3^a^	−1.52^a^	−3.4^a^	−3.11
(2) Carvacrol (L)	0.14^a^	−0.24^a^	0.49^a^	−0.13	2.12	7.19^a^
Carvacrol (Q)	0.14^a^	−0.04	−0.19^a^	−0.23	1.15	1.78
(3) Time (L)	−0.48^a^	0.09	2.06^a^	1.07	−0.22	6.95^a^
Time (Q)	0.02	−0.12^a^	−0.35^a^	1.26^a^	0.57	0.59
1L by 2L	−0.06	−0.02	0.06	−0.11	−0.39	−2.58
1L by 3L	−0.27^a^	−0.14^a^	0.57^a^	−0.54	−1.01	−4.52^a^
2L by 3L	−0.01	0.03	−0.03	0.63	0.59	−1.2
*R* ^2^_Model_	.78	.89	.97	.51	.29	.88

L, linear effect; Q, quadratic effect; XL by YL, interaction between factor X and factor Y.

a Factor with a significant effect on the response (*p* ≤ .05).

**Figure 5:**
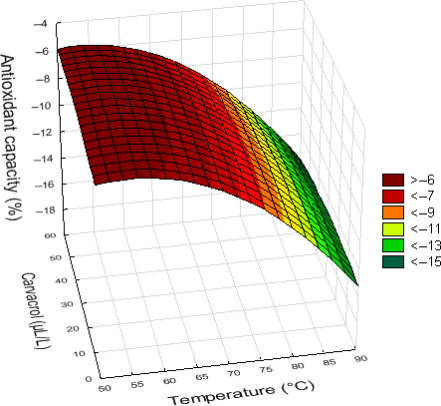
Effect of treatment temperature and concentration of carvacrol supplemented on the antioxidant capacity of pineapple juice (*t* = 20 min)

The assessment of the AOC of nontreated juices showed that pineapple juice (4.38 ± 0.77 mmol/L TEAC) had an AOC two and ten times higher than the ones of orange (2.29 ± 0.68 mol/L TEAC) and watermelon (0.42 ± 0.14 mmol/L TEAC) juices, respectively. From data obtained after heat treatment for this nutritional parameter, the statistical analysis generated a tendency explaining 88% of the ones obtained with watermelon juice (*R* ^2^ = .88), but only 51% and 29% of those obtained with pineapple and orange juices, respectively. The temperature of treatment had a significant linear negative effect on this AOC of the three fruit juices. This could be appreciated through the decrease observed with the rise of temperature. Besides, a quadratic negative effect was also observed with orange and pineapple juices. In fact, for these two latter juices, the effect of temperature on AOC was relatively small between 50 and 70°C, while AOC decreased almost linearly with temperature above 80°C (Figure 5). Carvacrol had a significant effect on the AOC only in treated watermelon juice, and this was linear and positive. The higher the level of carvacrol in watermelon juice, the higher its AOC. The length of the treatment significantly affected the AOC of juices with the exception of orange juice. A quadratic positive effect was observed with pineapple juice. Indeed, almost no change in AOC was noticed in this juice at the beginning of treatments, a stability that was followed by a little increase with time. In watermelon juice, treatment duration had a significant linear positive effect on this parameter. The higher the length of treatment, the higher its AOC, especially between 50 and 70°C. A negative interaction between time and temperature was also noticed in this juice.

**Figure 6:**
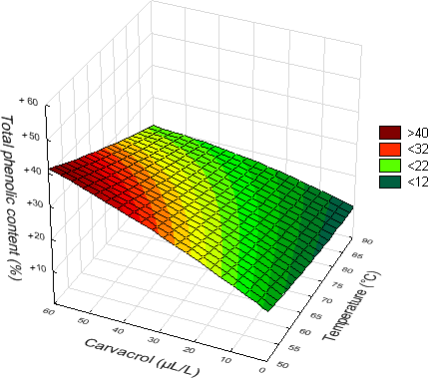
Effect of treatment temperature and concentration of carvacrol supplemented on the total phenolic content of watermelon juice (*t* = 20 min)

Data on the change in total phenolic and ascorbic acid contents of fruit juices after the different treatments are presented in Table 3, and the associated statistical analysis is in Table 4. TPC of nontreated orange (967 ± 139 mg/L GAE) and pineapple (995 ± 72 mg/L GAE) juices was almost similar but clearly higher than the one of watermelon juice (276 ± 31 mg/L GAE). From the CCD results, it came out that the temperature of treatment had a significant effect on fruit juices TPC. A linear positive effect was observed in the case of orange juice, but negative in the case of pineapple and watermelon juices. This could be appreciated through the increase in TPC with the rise of temperature for orange juice, and rather a decrease with the two other juices. Carvacrol supplementation had a linear positive significant effect on the TPC of the three juices. This was particularly obvious at high temperature for orange juice. Besides, a significant positive interaction with temperature was noticed in this juice. On the contrary, it is a negative interaction between carvacrol and temperature that was observed in watermelon juice. Indeed, the increase in temperature rather tended to reduce the positive impact of carvacrol (Figure 6). A significant effect of length of treatment was noticed only with watermelon juice, and this was quadratic. This could be seen through the rise of the TPC in samples treated for 10 and 20 min, an increase that was followed by a gradual reduction with time (samples treated for 30 and 40 min).

**Table 3:** Change in the total phenolic and ascorbic acid contents of fruit juices after the different treatments

Treatment conditions			Variation of the TPC (%)			Variation of the AAC (%)
Temperature (°C)	Carvacrol (μl/L)	Time (min)	Pineapple juice	Orange juice	Watermelon juice	Orange juice
90	0	0^a^	−1.34 ± 0.72	+2.49 ± 1.32	+4.53 ± 9.79	−8.35 ± 0.00
90	0	20	−4.54 ± 1.74	−0.94 ± 1.23	+7.34 ± 0.50	−15.45 ± 0.59
90	30	20	−0.83 ± 0.89	+4.68 ± 0.87	+13.18 ± 3.42	−17.54 ± 1.18
90	0	40	−4.14 ± 1.82	+1.83 ± 0.51	+8.86 ± 2.23	−21.72 ± 0.00
90	60	40	+0.31 ± 0.36	+9.56 ± 0.61	+24.40 ± 5.69	−21.72 ± 0.00
80	15	10	+4.45 ± 5.61	+5.60 ± 2.90	+13.67 ± 0.78	−11.69 ± 0.00
80	45	10	+2.08 ± 5.47	+6.81 ± 0.80	+22.94 ± 3.62	−11.69 ± 0.00
80	15	30	−1.05 ± 0.68	+4.03 ± 1.98	+14.17 ± 3.35	−15.04 ± 0.00
80	45	30	+9.91 ± 1.32	+4.73 ± 0.55	+27.97 ± 8.20	−12.53 ± 1.18
70	0	0^a^	−1.61 ± 1.25	+1.70 ± 0.67	+4.50 ± 9.29	−2.99 ± 0.00
70	30	0^a^	+2.26 ± 0.97	+2.11 ± 1.51	+14.15 ± 1.84	−2.99 ± 0.00
70	0	20	−2.96 ± 1.36	+1.11 ± 2.89	+11.73 ± 0.98	−5.24 ± 1.06
70	30	20	+2.06 ± 2.33	+0.78 ± 4.11	+25.65 ± 7.95	−6.73 ± 1.06
70	30	20	+1.82 ± 2.39	+2.13 ± 3.86	+13.79 ± 1.63	−4.49 ± 0.00
70	30	20	+1.41 ± 1.91	+1.29 ± 0.88	+26.90 ± 1.77	−4.11 ± 0.53
70	60	20	+3.69 ± 2.38	+4.52 ± 3.42	+25.54 ± 3.03	−5.61 ± 0.53
70	0	40	−2.70 ± 4.26	−0.10 ± 2.19	+7.97 ± 5.60	−8.97 ± 0.00
70	30	40	−0.25 ± 2.71	+0.33 ± 1.47	+15.82 ± 4.10	−8.97 ± 0.00
60	15	10	+9.33 ± 0.39	+1.98 ± 0.36	+31.21 ± 4.47	−1.83 ± 0.86
60	45	10	+9.07 ± 4.83	+2.75 ± 0.82	+31.87 ± 7.86	−3.36 ± 0.43
60	15	30	+6.04 ± 2.68	−0.53 ± 0.68	+24.94 ± 7.11	−4.89 ± 0.00
60	45	30	+6.56 ± 1.53	+4.00 ± 0.46	+22.76 ± 1.21	−4.89 ± 0.00
50	0	0^a^	+0.03 ± 2.51	+1.45 ± 2.79	+6.11 ± 11.12	0.00 ± 0.00
50	0	20	+3.82 ± 5.13	−3.68 ± 1.16	+17.65 ± 5.49	−1.03 ± 0.49
50	30	20	+4.43 ± 5.00	−0.01 ± 1.82	+28.42 ± 3.59	−0.69 ± 0.00
50	0	40	+0.86 ± 3.11	−2.68 ± 0.80	+5.69 ± 4.90	−3.77 ± 0.49
50	60	40	+9.67 ± 1.84	−0.68 ± 1.75	+42.45 ± 3.67	−3.09 ± 0.49
**Nontreated juice**			**TPC (mg/L GAE)**			**AAC (mg/L)**
			995 ± 72	967 ± 139	276 ± 31	299 ± 40

GAE, gallic acid equivalent; TPC, total phenolic content; AAC, ascorbic acid content.

a Samples were removed from the water bath as soon as they reached the desired temperature.

**Table 4:** Effect estimates of process variables on the change in total phenolic and ascorbic acid contents of fruit juices observed

Factor	Effect on total phenolic content			Effect on ascorbic acid content
	Pineapple juice	Orange juice	Watermelon juice	Orange Juice
(1) Temperature (L)	−3.16^a^	2.76^a^	−6.44^a^	−7.71^a^
Temperature (Q)	0.18	−0.21	0.4	−1.54^a^
(2) Carvacrol (L)	2.96^a^	2.38^a^	9.25^a^	−0.61
Carvacrol (Q)	−0.81	0.04	−0.9	0.21
(3) Time (L)	−0.33	−0.93	2.25	−2.86^a^
Time (Q)	−0.47	0.29	−2.62^a^	−0.2
1L by 2L	−0.24	0.62^a^	−1.93^a^	−0.24
1L by 3L	−0.28	0.39	0.96	−0.9
2L by 3L	0.35	0.14	1	0.48
*R* ^2^_Model_	.48	.61	.64	.95

L, linear effect; Q, quadratic effect; XL by YL, interaction between factor X and factor Y.

a Factor with a significant effect on the response (*p* ≤ .05).

**Figure 7:**
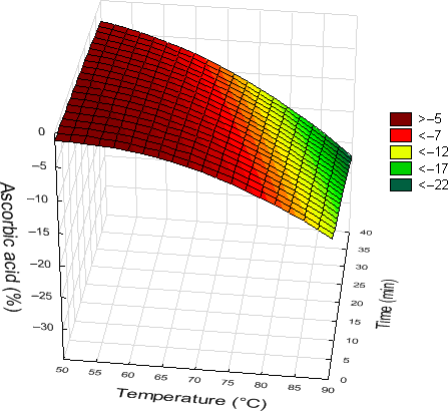
Effect of temperature and length of treatment on the ascorbic acid content of orange juice (carvacrol = 30 μl/L)

Concerning AAC (vitamin C content), temperature of treatment had a significant linear and quadratic negative effect on this parameter (*R* ^2^ = .95). The rise of temperature led to a loss of the AAC that was higher at 80 and 90°C, than between 50 and 70°C. Time also had a significant negative effect, the losses increasing with the length of treatment. The statistical analysis also revealed a negative interaction between treatment duration and temperature. In fact, the negative impact of the length of treatment on juice AAC was more important above 80°C than below (Figure 7). No significant effect of carvacrol was noticed on this nutritional parameter.

## 4 DISCUSSION

Color is one of the most important quality attributes of fruit juices. It determines acceptability by consumers and purchase decisions (Cortés et al., 2008; Meléndez‐Martínez, Gómez‐Robledo, Melgosa, Vicario, & Heredia, 2011; Wibowo et al., 2015). The only carvacrol supplementation did not change the color of fruit juices. The combined treatment also had almost no impact on orange juice color. This color of orange juice has been correlated to its carotenoid pigment contents and is generally used as an index of this carotenoid content (Meléndez‐Martínez et al., 2011; Vikram et al., 2005; Wibowo et al., 2015). Sánchez‐Moreno et al. (2005) observed that thermal treatment of orange juice at 70°C for 30 s and 90°C for 1 min had no impact on carotenoid contents. As reported by Gama and de Sylos (2007), carotenoids are generally stable to heat treatment such as blanching, cooking, and canning. These authors found that total carotenoid pigment content loss in Valencia orange juice was not significant after a thermal pasteurization at temperatures between 95 and 105°C for 10 s. If certain carotenoid compounds were negatively affected by this treatment (violaxanthin and lutein), this was not the case for other compounds such as β‐carotene, α‐carotene, and β‐cryptoxanthin. In the case of pineapple juice, we noticed that temperature and length of treatment had a significant effect on visual color, but this was very weak. Thermal treatment of pineapple juice at high temperature has been reported as leading to changes in color due to nonenzymatic browning through Maillard reaction, pigment destruction, and sugar caramelization. By treating this juice between 55 and 95°C, Rattanathanalerk, Chiewchan, and Srichumpoung (2005) observed a linear increase in browning indicators (hydroxymethylfurfural and brown pigments) with temperature and a reduction in total carotenoids (pigments found in pineapple). In contrast to the two other juices, the color of watermelon juice changed more remarkably under the effect of the combined treatment. The rise of temperature and length of treatment significantly increased its hue angle value, which means a decoloration of the juice naturally red to a more yellowish color. Sharma, Kaur, Oberoi, and Sogi (2008) had already observed, by treating watermelon juice between 50 and 90°C up to 5 hr, that the degradation of total carotenoids, lycopene, and color was following a first‐order kinetic. They found a clear relationship between lycopene, total carotenoid content, and visual color of watermelon. In fact, lycopene is the component that gives to watermelon its red color. It can represent up to 75% of some watermelon cultivars (Oms‐Oliu, Odriozola‐Serrano, Soliva‐Fortuny, & Martín‐Belloso, 2009). The destruction of this compound under the effect of the process may therefore explain the high important color changes observed.

Antioxidant activity, TPC, and vitamin C (ascorbic acid) content are three key parameters to evaluate nutritional quality and potential health benefits of fruit juices. Orange juice is a very popular and rich source of vitamin C (Vikram et al., 2005), a thermolabile compound which serve as an indicator of the losses of other vitamins (Chakraborty, Rao, & Mishra, 2015; Torregrosa, Esteve, Frígola, & Cortés, 2006; Van Den Broeck, Ludikhuyze, Weemaes, Van Loey, & Hendrickx, 1998). Its degradation in fruit juices through thermal processing follows a first‐order kinetic (Paul & Ghosh, 2012; Sánchez‐Moreno, Plaza, De Ancos, & Cano, 2006; Sánchez‐Moreno et al., 2005; Vikram et al., 2005). This corroborates with the negative significant effects of temperature and treatment duration noticed on AAC in this study.

In contrast to pineapple and watermelon juices, orange juice showed a TPC which increased with temperature. Similar results were obtained by Lo Scalzo, Iannoccari, Summa, Morelli, and Rapisarda (2004) who treated blood orange juice at 80°C and observed an increase in its main phenolic substances such as anthocyanins and total cinnamates. As hypothesized by these authors, the thermal treatment may induce liberation of molecules previously complexed or polymerized and retention of active principles caused by the inactivation of the enzymes involved in their catabolism. This hypothesis may also be true in the case of pineapple and watermelon juices where we observed at mild temperatures an increase in TPC, which rather decreased with the rise of temperature. This therefore suggests a difference in the phenolic profile (nature of compounds) of fruit juices, those present in one being more thermosensitive than those present in the others.

Ascorbic acid, phenolic compounds, and carotenoids are compounds that confer the AOC of fruit juices. Many authors have described the negative effect of thermal treatments on this AOC of fruit juices. In the case of orange juice, vitamin C has been reported to account for 65%–100% of its AOC, contribution of carotenoids being very negligible (Gardner et al., 2000). A correlation can therefore be made between the high decrease in AOC and the AAC losses observed at 90°C. The AOC of pineapple juice has rather been reported to be mainly associated with its TPC (Almeida et al., 2011), vitamin C conferring only 5% of this capacity (Gardner et al., 2000). The significant negative effect of temperature noticed on the AOC of this juice could therefore be correlated to the significant negative effect on its TPC. Concerning watermelon, it is known as containing very low vitamin C and TPCs. As noticed in this study, AOC of nontreated watermelon juice was the lowest of the three fruit juices (100%) produced. Oms‐Oliu et al. (2009) observed that AOC of watermelon juice was linked to its lycopene content. The high reduction in this compound under the effect of the thermal treatment (appreciable through the change in juice color) may therefore explain the decrease in its AOC at high temperatures. A link can also be established between the increase in TPC and the increase in AOC observed in this juice at mild temperatures.

The supplementation of fruit juices with carvacrol had a positive effect on their TPC after thermal treatment as it is a phenolic compound (5‐isopropyl‐2‐methylphenol). At the tested concentrations, this supplementation did not have an impact on AOC of pineapple and orange juices, but on the one of thermally treated watermelon juice where the initial AOC was very low. This suggests, considering the almost null AOC of the tested concentrations, that carvacrol may interact during the thermal treatment with some fruit juices components leading to the formation of compounds with an AOC. This was more appreciable in watermelon juice as it had an almost null AOC.

## 5 CONCLUSIONS

Supplementation of fruit juices with natural antimicrobials like carvacrol followed by a mild thermal treatment may definitely be an effective alternative to conventional pasteurization in order to ensure their safety while preserving their color and nutritional value. However, the application of such combined treatment must take into account the nature of the fruit juice to be treated as their properties or characteristics are not the same.

## CONFLICT OF INTEREST

The authors declare that they do not have any conflict of interest.

## ETHICAL STATEMENT

This study does not involve any human or animal testing.
